# The recurrence of prolactinoma after withdrawal of dopamine agonist: a systematic review and meta-analysis

**DOI:** 10.1186/s12902-021-00889-1

**Published:** 2021-11-13

**Authors:** Yunzhi Zou, Depei Li, Jiayu Gu, Siyu Chen, Xia Wen, Jiajun Dong, Xiaobing Jiang

**Affiliations:** 1 Department of Neurosurgery/Neuro-oncology, Sun Yat-sen University Cancer Center. State Key Laboratory of Oncology in South China, Collaborative Innovation Center for Cancer Medicine, Guangzhou, 510060 China; 2 Jiangmen Central Hospital, Affiliated Jiangmen hospital of Sun Yat-Sen University, Jiangmen, 519000 China

**Keywords:** Prolactinoma, Dopamine agonist, Cabergoline, Bromocriptine, Recurrence, Meta-analysis

## Abstract

**Background:**

Prolactinoma is the major cause of hyperprolactinemia, and dopamine agonists (DAs) are generally the first-line treatment for them. Several studies have reviewed the recurrent rate of hyperprolactinemia after DAs withdrawal. However, few of them have concerned the recurrence risk of prolactinoma following the withdrawal of DAs.

**Methods:**

Three medical databases, PubMed, EMBASE and Cochrane library, were retrieved up to February, 14, 2021 to identify studies related to recurrence of prolactinoma and withdrawal of DAs. Statistical analyses including meta-analysis, sensitivity analysis, meta-regression, funnel plot and Egger test were performed through software R.

**Results:**

A total of 3225 studies were retrieved from the three data bases, and 13 studies consisted of 616 patients and 19 arms were finally included in this systematic analysis. There was no significant heterogeneity among the included studies, and fixed effect model was thus used. The pooled recurrence proportion of prolactinoma after withdrawal of DA was 2% with a 95% confidence interval (CI) of 1–3%.

**Conclusion:**

Our study showed a very low recurrent rate of prolactinomas after DAs withdrawal. Much more prospective studies with larger cases and longer follow-up period are encouraged to confirm our finding.

**Trial registration:**

**Registration number** CRD42021245888 (PROSPERO).

## Background

Pituitary adenomas are one of the most common intracranial tumors, and approximately half of these tumors are hormone-secreting [1]. Prolactinoma are the most common pituitary adenomas, accounting for 70–80% of all endocrine-secreting pituitary adenomas. The incidence of new-diagnosed prolactinoma is about 3–5 per 100,000 population per year [2]. Prolactinoma is the major cause of hyperprolactinemia, which frequently induces amenorrhea, galactorrhea and infertility in female and erectile dysfunction in male [3]. Macroadenomas may cause additional symptoms related to mass effects on adjacent neurovascular structures [1].

Dopamine agonists (DAs), including bromocriptine (BRC) and cabergoline (CAB), are first-line treatments for most of prolactinoma. DAs are effective in normalizing prolactin levels (68% of patients), reducing tumor size (62% of patients) and relieving infertility (53%) and other symptoms [4]. The recommended duration of DA treatment for prolactinoma is at least two years until normo-prolactinemia and tumor disappearance [4]. Of note, the recurrence of hyperprolactinemia after withdrawal of DAs is higher than expected, which is reported as 30–80% [5–8] according to the type of DAs, treatment duration and tumor size [5, 6]. The remission rate was better in patients using CAB and those with microadenomas. However, the proportion and risks of tumor enlargement after DAs withdrawal has been seldom concerned before. To address this issue, we performed a systematic review and meta-analyses to investigate the rate of prolactinoma recurrence after DAs withdraw. In the present study, we found a very low recurrent rate of 2%, when the DAs was ceased.

## Methods

### Protocol and registration

The protocol of this systematic review and meta-analysis was uploaded on PROSPERO with registration number # CRD42021245888.

### Information sources

Three medical databases, including PubMed, Cochrane library and EMBASE, were used to retrieve literature. Retrieval was restricted by studies published before February 14, 2021. The related references of other reviews were also included.

### Search

The search terms are “(Bromocriptine withdraw) OR (Bromocriptine withdrawal) OR (Bromocriptine discontinue) OR (Bromocriptine discontinued) OR (Bromocriptine discontinuance) OR (Cabergoline withdraw) OR (Cabergoline withdrawal) OR (Cabergoline discontinue) OR (Cabergoline discontinued) OR (Cabergoline discontinuance)”.

### Study selection

Studies that refereed to prolactinoma recurrence after withdrawal of DAs were retrieved and loaded into Reference management software NoteExpress 3.2.0.7276 (AegeanSoft Corporation). There is no limitation on the language type of included studies. Duplicate studies were checked through software NoteExpress. Preliminary screening was performed based on title, abstract and keywords thereafter. Then we screened remaining studies based on inclusion and exclusion criteria by looking at full text.

All authors related to study selection process were divided into two groups. Y. Zou and D. Li were in group A. S. Chen, J. Gu and X. Wen were in group B. Group A and Group B screened the retrieved papers separately by reviewing the titles, abstracts, and keywords. Disagreements were solved by discussion within two groups. Any disagreements without consensus with discussed with experimental researcher (X. Jiang).

The inclusion criteria are as follows:

i. Participants were prolactinoma patients;
ii. Participants were older than 18;
iii. Duration of DAs treatment was at least 3 months, and normoprolactinemia combined with significant tumor shrinkage or disappearance had to be attained during the treatment;
iv. The main drugs used in study were BRC or CAB;
v. Prolactinoma enlargement or recurrence after DAs withdrawal must be reported or can be calculated;
vi. Patient mean follow-up period was at least 3 months;
vii. There should be no duplicated cohorts. Therefore, if duplicated cohorts were presented, the largest one will be included.

The exclusion criteria are as follows:

i. Participants were pregnant;
ii. The normal reference values of prolactin were not reported;
iii. The proportion of pre-intervention including radiotherapy and surgery was more than 20% [5];
iv. The proportion of participants lost to follow-up was more than 20% [9, 10];
v. If duplicated cohorts were presented, the smaller cohorts will be removed;
vi. Studies induced high heterogeneity among all studies.

### Data collection process

Group A and Group B extracted related data separately into Excel table. Disagreements were solved by discussion within two groups. Any disagreements without consensus were discussed with experimental researcher (X. Jiang).

### Data items

We extracted following data: study design, etiology, drug types, drug dosage, treatment duration, number of patients, age of patients, sex ratio, intervention before medication, detection method of tumor, hormone measurement methods, tumor diameter before treatment, PRL (prolactin) level before treatment, regression of tumor before withdrawal, normalization of serum PRL before withdrawal, prolactinoma recurrence after withdrawal, follow-up time.

### Summary measures

Recurrence proportion was outcome indicators in individual studies. Pooled recurrence proportion and 95% confidential interval (CI) were effect size in this systematic review and meta-analysis.

### Synthesis of results

Two main units of PRL level (mIU/l and ng/ml) were used in included studies. In this systematic review and meta-analysis, nanograms per milliliter was used as the units of PRL level. The conversion factor between milliunits per liter and nanograms per milliliter were 30 [8]. The unit of prolactinoma diameter was millimeter. The recurrence proportion of prolactinoma after withdrawal was calculated and pooled. It is presented using proportion with 95% CI. In order to increase the credibility and authenticity of the results, we evaluated heterogeneity among included studies using I^2^ test and χ2 statistic. If *P* < 0.1 or I^2^ > 50%, this review will be significant heterogeneity. Then, random effect model will be used; otherwise, fixed effect model will be used. Heterogeneity was analyzed through sensitivity analysis and meta-regression. Sensitivity analysis omits each study one by one and detects the change of heterogeneity. Meta-regression detected the possible sources of heterogeneity separately with statistically significant *P* value. Publication bias was detected and analyzed through funnel plot and Egger text. Funnel plot is a qualitative method with an acceptable region. Egger text is a quantitative method with statistically significant *P* value. P<0.05 indicates statistically significant publication bias. Statistical analysis was performed through R version 4.0.5.

## Results

### Study selection

**Fig. 1:**
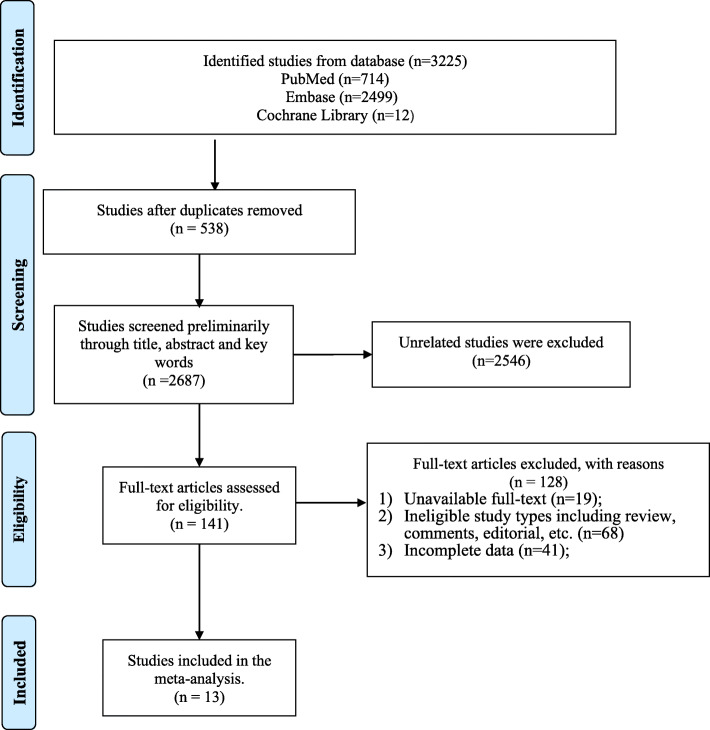
Flow diagram of the studies included in this systematic review and meta-analysis

Flow diagram of literature retrieval was shown in Fig. 1. A total of 3225 studies were retrieved, including 714 from PubMed, 2499 form Embase and 12 from Cochrane library. 538 of them were removed due to duplication and 2546 were excluded through preliminary screening. The remaining 141 studies were screened through full-text assessment thereafter. In the process of full-text assessment, 19 were excluded due to unavailable full-text, 68 were excluded due to ineligible study types and 41 were excluded due to incomplete data. Finally, 13 studies were included in this systematic review and meta-analysis.

### Study characteristics

There were 19 arms and 616 patients in thirteen studies, which were all single-armed studies (Table 1) [7, 11–22]. The minimal number of participants in individual study was 6 [11, 13]. The maximum number of participants in individual study was 194 [21]. In term of year of publication, six arms [11, 13, 15, 16] were earlier than the year 2000 and others [7, 12, 14, 17–22] were later than the year 2000. In term of study area, four arms [19, 22] were from America (including Northern America and Southern America), three arms [7, 17, 20] were from Asia and twelve arms [11–16, 18, 21] were from Europe. Patients included were all prolactinomas complicated with hyperprolactinemia. Based on the size of prolactinoma, eight arms [11, 13–15, 18, 19, 21, 22] were microprolactinoma, eight arms [14, 15, 17–22] were macroprolactinoma and three arms [7, 12, 16] had no detail. The age of patients was ranged from 20 to 60 with mean age ranged from 30 to 40. 367 of the them were female. Eight arms [12, 13, 15, 17, 18, 20] did not give the detail of sex ratio. Interestingly, most of female were microprolactinoma, while most of male were macroprolactinoma. In term of intervention before treatment, twelve arms [7, 11, 14, 15, 17, 18, 20] had no pre-intervention, five arms [12, 16, 19, 22] had pre-intervention and two arms [13, 21] had no details. In Johnston D G’s study, two patients had previously been treated surgically [16]. In Kharlip J’s study published in 2009, eight patients were with microprolactinoma and four with macroprolactinoma had BRC treatment before [22]. In V.Q. Passos’s study published in 2002 and Sala E’s study published in 2016, authors only mentioned the presence of pre-intervention [12, 19]. In term of types of DAs, three arms [11, 16, 17] only used BRC, eleven arms [12, 14, 15, 20–22] only used CAB, and five arms [7, 18, 19] used both BRC and CAB. Besides, quinagolide was included in two arms [18]. In term of treatment duration, two arms [13, 16] were shorter than 24 months, twelve arms [7, 11, 12, 14, 15, 20–22] were longer than 24 months and five arms [17–19] had no details. In term of tumor detection, two arms [11, 13] used computed tomography (CT), eleven arms [7, 14–17, 20–22] used MRI, three arms [12, 19] used both CT and MRI and one arm did not give detail information. In term of PRL measurement method, five arms [17, 21, 22] did not give detail information, while the others [7, 11–16, 18–20] used immunoassay. The tumor diameter and PRL concentration before treatment were shown in Table 1. Tumors were all shrank significantly (more than 50%) after treatment. In term of follow-up duration (Table 1), four arms [14, 16, 22] were shorter than 12 months, six arms [11, 12, 15, 17, 20] were between 12 months and 24 months, and nine arms [13, 14, 18, 19, 21] were longer than 24 months.

**Table 1:** The characteristics of included studies

First Author	Year	Region	No. patients	Drug types and dosage	duration (months)	age (yr)	M/F	Pre intervention	Tumor detection	PRL measurement methods	Initial tumor diameter (mm)	Initial PRL concentration (ng/ml)	Regression of tumor	Normalize serum prolactin before withdrawal	Prolactinoma recurrence (n)	Follow-up (months)
Moriondo, P [11]	1985	Italy	6	BRC: 4 was 10 mg/d 2 was 5 mg/d	24	Mean: 35.33 SEM: 5.07	0/6	No	CT	RIA	NA	Mean: 84.5 SEM: 22.7	All	All but one	1	16
Cannavo` S [15]	1999	Italy	9 MAC 18 MIC	“A”	24	Mean ± SD (range) MAC: 28.9 ± 3.2 (18–45) MIC: 29.6 ± 2.0 (18–62)	NA	No	MRI	IA	Mean ± SEM. MAC: 15.64 ± 3.98 MIC: 6.58 ± 1.96	Mean ± SEM MAC: 404.09 ± 281.53 MIC: 192.88 ± 112.35	Mean ± SEM (*p* value) MAC: 6.9 ± 1.8 mm (p < 0.001) MIC: 3.0 ± 0.5 mm (*p* < 0.001)	All but three	0	12
Johnston, D G [16]	1984	UK	15	BRC: 7.5-20 mg daily (9 was 20 mg daily)	Mean: 44.4 SEM: 4.68	Mean: 40.87 SEM: 3.29	8/7	2 with surgery before	CT	RIA	NA	Range: 49.1–38,333	All	All but one	1	Mean: 3.14 SEM: 0.66
Wu, Z B [17]	2008	China	14	“B”	NA	Mean: 36 SEM: 12	NA	No	MRI	NA	Mean: 45 Range: 20–97	NA	All	All	0	Mean 18 (at least 3 mo)
Anagnostis, P [18]	2012	Greece	20 MIC 6 MAC	CAB & BRC	NA	Mean ± SEM MIC: 32 ± 1 MAC: 41 ± 3 Total: 35 ± 2	ND	No	NA	ICMA	Mean ± SEM MIC: 7.4 ± 1 MAC: 24.1 ± 0.39 Total: 13.7 ± 2	Mean ± SEM MIC: 112 ± 19 MAC: 263 ± 59 Total: 165 ± 26	Mean ± SEM (mm) MIC: 1.6 ± 0.6 MAC: 6 ± 4.2 Total: 2.6 ± 0.7	Mean: 12.2 SEM: 2.3 Range: 0.5–44.7	2 (1 MIC and 1 MAC)	Mean: 79 SEM: 11 Range: 12–240
V.Q. Passos [19]	2002	Brazil	16 MIC 11 MAC	BRC (mg/week) median: 5 range: 1.25–25	NA	Median 28 Range 15–31 Mean 29.85 SD 8.94	4/23	Yes	CT & MRI	RIA	NA	Median: 257 Range: 36–2000 Mean: 417 SD: 473	NA	All	0	Median 44; Range 3–240.
Watanabe, S [20]	2017	Japan	11	CAB (mg/week) Maximum: 1 (range 0.25–12); Maintenance: 0.5 (range 0.25–3.0).	60	Mena: 36 range: 16–64	ND	No	MRI	IA	Mean: 21 Range: 10–76	Mean: 486.9 Range: 1–23,500	All	Unit: ng/ml Mean: 7.2 Range: 1.0–71.9	0	12
Colao, A [21]	2007	Italy	115 MIC 79 MAC	CAB (mg/week) mean ± SD (median) MIC: 1.2 ± 0.5 (1.0) MAC: 1.2 ± 0.4 (1.0)	mean ± SD (median) MICs: 43 ± 15 (45) MACs: 42 ± 13 (36)	Mean ± SD (median) MIC: 32 ± 11 (29) MAC: 44 ± 15 (46)	MIC: 12/103 MAC: 36/43	NA	MRI	NA	Mean ± SD (range) MIC: 6.8 ± 1.5 (3.3–10) MAC: 17.2 ± 6.2 (10.3–50)	Mean ± SD (range) MIC: 157.2 ± 50 (67–300) MAC: 891.67 ± 1341 (197–9715)	Mean ± SD (range)(mm): MIC: 1.5 ± 2.0 (0–5.6) MAC: 2.4 ± 3.5 (0–9.5)	Mean ± SD (range)(ng/ml) MIC: 24.8 ± 17.93 (3–78) MAC: 31.6 ± 22.3 (10.3–90)	0	Mean ± SD (median) MIC: 47 ± 29 (48) MAC: 44 ± 28 (48)
Kharlip, J [22]	2009	USA	31 MIC 11 MAC	“C”	Median (range) MIC: 43 (23–119) MAC: 56 (26–205)	Median (range) MIC: 44 (18–68) MAC: 54 (36–75)	MIC: 5/26 MAC: 7/4	8 MIC & 4 MAC had BRC before	MRI	NA	Median (range) MIC: 8 (5–10) MAC: 15 (11–29)	Mean (range) MIC: 73 (27.2–182.3) MAC: 310 (103–1122)	Mean (range)(mm) MIC: 3 (0–9) MAC: 0 (0–7)	Median (range)(ng/ml) MIC: 3.6 (0.1–17) MAC: 1.9 (0.1–6.2)	0	3
Sala, E [12]	2016	Italy	32	CAB (mean ± SD) (1) Week dosage (mg): MIC: 0.96 ± 0.41 MAC: 0.97 ± 0.37 (2) Total amount (mg): MIC: 397.4 ± 274.6 MAC: 313 ± 198.6	Mean ± SD: MICs: 66 ± 39.6 MACs: 79.2 ± 30	Mean ± SD MIC: 42.4 ± 9.9 MAC: 70 ± 10.9	NA	Yes	CT & MRI	FIA	NA	Mean ± SD MIC: 113.5 ± 54.12 MAC: 258.9 ± 211.3	76% in MIC; 92% in MAC.	mean ± SD (ng/ml) MIC: 7.3 ± 4.3 MAC: 13.4 ± 14.1	0	12
Muratori, M [13]	1997	Italy	6	“D”	12	Range 25–48	NA	NA	CT	IFMA	NA	Mean ± SEM 89.83 ± 12.76	All	Unit: ng/ml Mean: 9 SEM: 1.81	1	Mean: 52.83 SEM: 4.53
Dogansen, S C [7]	2016	Turkey	36	“E”	Mean ± SD (range) 66.7 ± 30.4 (27–138)	Mean: 32.1 SD: 10.9 Range: 17–66	12/24	No	MRI	IA	Mean: 19.4 SD: 12.4 Range: 6–52	Mean: 2303 SD: 4190 Range: 112–18,500	Disappearance (n, %): 11 (31%) mean ± SD (range) Diameter (mm): 3.3 ± 3.1 (0–10) Percentage of tumor shrinkage (%): 76.5 ± 18.6 (50–100)	mean ± SD (range) PRL level (ng/ml): 8.4 ± 6.7 (0.4–21) Percentage reduction (%): 95.1 ± 5.3 (81–99)	0	Mean: 28.6 Range: 7–26
Colao, A [14]	2003	Italy	105 MIC 70 MAC	CAB (mg/week) Median (range) MIC: 1 (0.5–3.5) MAC: 1 (1–2)	Median (range) MIC: 48 (24–75) MAC: 42 (24–72)	Median (range) MIC: 30 (15–66) MAC: 40 (19–70)	MIC: 11/94 MAC: 33/37	No	MRI	RIA	Mean ± SD. MIC: 6.9 ± 1.6; MAC: 17.1 ± 6.4.	Mean ± SD MIC: 162.2 ± 48.2 MAC: 915.6 ± 1413	Mean ± SD (mm) MIC: 1.2 ± 1.6 MAC: 2.3 ± 3.3	Mean ± SD (ng/ml) MICs: 6.0 ± 5.2 MAC: 5.3 ± 3.0	0	At least 24 months

M/F: Male/Female

SD: standard deviation; SEM: standard error of mean

MIC: microprolactinoma; MAC: macroprolactinoma; PRL: prolactin

BRC: bromocriptine; CAB: cabergoline

IA: Immunoassay; RIA: radioimmunoassay; FIA: fluorimmunoassay; ICMA: Immunochemiluminencent assay; IFMA: Immunofluorimetric assay

“A”: 0.25 mg CAB twice a week for 4 weeks. The dose was increased stepwise in 0.5 mg increments until reaching lowest maximally effective and tolerated dose

“B”: Started with BRC 2.5 mg/d. Increased to 7.5 mg/d within 2 or 3 weeks. Increased to 15 mg/d if serum PRL levels were not controlled. Reduced to no more than 5.0 mg/d once prolactin level was within the normal range for 3 months

“C”: CAB (mg/week). Max dose and number of patients: MIC: 0.25 (3), 0.5 (9), 1.0 (17), 1.5 (2), MAC: 0.25 (0), 0.5 (2), 1.0 (7), 1.5 (2); Dose before stopping treatment and number of patients: MIC: 0.25 (13), 0.5 (7), 1.0 (9), 1.5 (1), missing (1), MAC: 0.25 (4), 0.5 (6), 1.0 (1), 1.5 (0)

“D”: Cabergoline. Started with 0.5 mg/week. Further dose increments by 0.25–0.5 mg. The maximum dose was 3 mg/week

“E”: Mean ± SD (range). Unit: mg/week. For CAB: Max dose: 0.8 ± 0.3 (0.5–1.5), Min dose before withdrawal: 0.3 ± 0.1 (0.25–0.5). For BRC: Max dose: 7.4 ± 4.3 (2.5–20), Min dose before withdrawal: 4.2 ± 2.6 (1.25–7.5)

### Statistical analysis

**Fig. 2:**
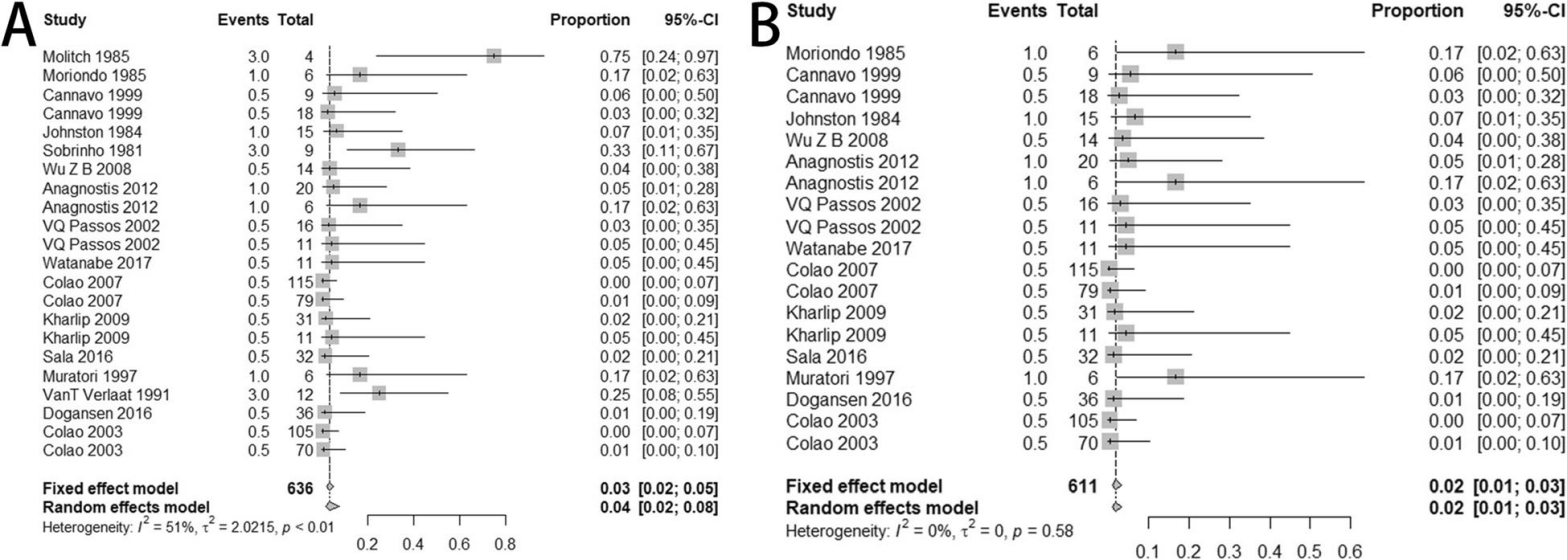
Pooled proportions of prolactinoma recurrence among included studies. **A**) Before correction; **B**) After correction

**Fig. 3:**
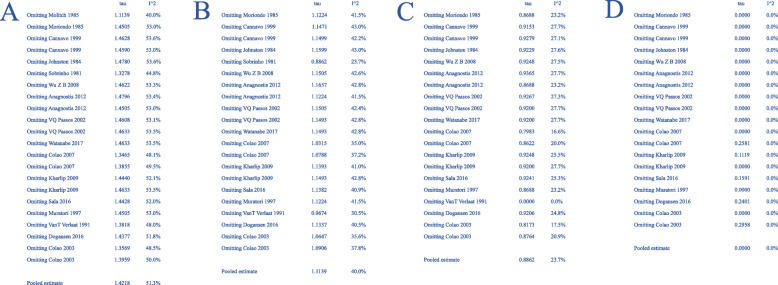
Sensitivity analysis. **A**) Before correction, Molitch’s study published in 1985 declined the value of I^2^ significantly; **B**) After omitting Molitch’s study published in 1985, Sobrinho’s study published in 1981 declined the value of I^2^ significantly; **C**) After omitting Sobrinho’s study published in 1981, VanT Verlaat’s study published in 1991 declined the value of I^2^ significantly; **D**) After correction

**Fig. 4:**
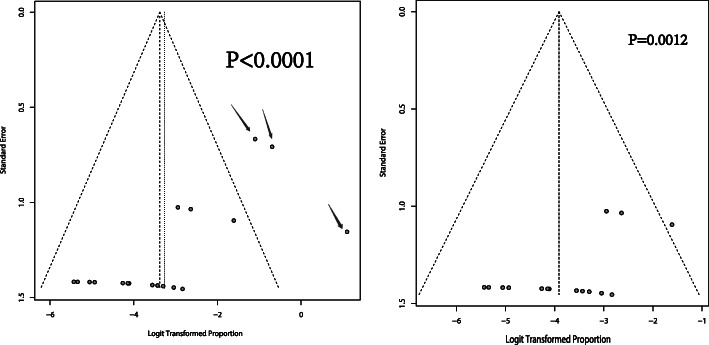
Funnel plots. **A**) Before correction; **B**) After correction

Before correction, the heterogeneity of included studies was significant (I^2^ = 51%, *p* < 0.01) (Fig. 2A). Therefore, synthesis of individual studies was performed through random effects model. The pooled recurrence proportion of prolactinomas after withdrawal of DAs was 4% (95% CI: 2–8%) (Fig. 2A). Through sensitivity analysis, three studies, Molitch’s study published in 1985, Sobrinho’s study published in 1981 and VanT Verlaat’s study published in 1991, declined the value of I^2^ significantly (Fig. 3A-C). Through meta-regression analysis, six factors including region of publication, year of publication, tumor size, drug type, treatment duration and follow up time were analyzed. As shown in Table 2, all the factors above did not significantly affect the analysis results. In terms of publication bias, three arms were out of acceptable range (Fig. 4A). The result of Egger test was less than 0.0001.

**Table 2:** Meta-regression analysis

	estimate	se	zval	pval	ci.lb	ci.ub
**Region of publication** (Europe vs. America vs. Asia)	−0.5757	2.4462	−0.2354	0.8139	−5.3702	4.2187
**Year of publication** (Before 2000 vs. after 2000)	−2.8189	1.4811	−1.9032	0.057	−5.7218	0.084
**Tumor size** (< 24 vs. ≥24 months)	−0.6388	1.0198	−0.6263	0.5311	−2.6376	1.3601
**Drug type** (bromocriptine vs. cabergoline)	−1.9056	1.5734	−1.2111	0.2258	−4.9895	1.1783
**Treatment duration** (< 24 vs. ≥24 months)	−1.0916	1.0284	−1.0614	0.2885	−3.1072	0.9241
**Follow-up time** (< 12 vs. 12–24 vs. < 24 months)	0.814	1.2451	0.6538	0.5132	−1.6263	3.2544

se: standard error; zval: z value; pval: *p* value; ci.lb.: Lower bounds of 95% confidential interval; ci.ub: Upper bounds of 95% confidential interval;

After correction, three studies including Molitch’s study published in 1985, Sobrinho’s study published in 1981 and VanT Verlaat’s study published in 1991 were removed. The heterogeneity among included studies was not statistically significant (I^2^ = 0% and *p* = 0.58) (Fig. 2B), and sensitivity analysis did not detect statistical significance thereafter (Fig. 3D). Therefore, fixed effect model was used. The correctly pooled recurrence proportion of prolactinoma after withdrawal of DAs was 2% (95% CI: 1–3%) (Fig. 2B). In terms of publication bias, one arm was out of acceptable range (Fig. 4B). The result of Egger test was 0.0012, which was less than 0.05.

## Discussion

Prolactinomas are the most common pituitary adenomas, with a prevalence as high as 45 per 71,000 inhabitants [23]. They are the major cause of hyperprolactinemia, which usually induces oligoamenorrhea, galactorrhea or infertility in women, and decreases sexual potency in men [3]. Dopamine agonists, including bromocriptine and cabergoline, remain the primary treatment choice for them. Several studies tried to stop the use of DAs when hyperprolactinemia was returned, and most of the cases were found to relapse. However, none studies have systematically explored the risk of tumor recurrence after DAs withdrawal. In the present study, we investigated the recurrent rate of prolactinomas after the stop of DAs. Meta-analysis showed a pooled recurrence proportion of prolactinoma after withdrawal of DAs was 2% (95% CI: 1–3%).

Prolactinoma is the most frequent cause of hyperprolactinemia. Although the majority of them cloud be controlled by DAs, patients have to take medicine life-timely. Several previous systematic studies have explored the possibility and timing of DAs withdraw, when hyperprolactinemia was relieved. Dekkers, et al. reported the pooled proportion of persisting normo-prolactinemia after DAs withdrawal was 21% [5]. Xia et al. reported a similar the proportion of persisting normoprolactinemia of 36.6% [6]. Different from these studies [5, 6, 8], we primarily concerned the treatment of DAs on prolactinoma, instead of hyperprolactinemia. We found a very low recurrent rate of prolactinomas, after the withdraw of DAs. This is a very interesting finding. If it is so, part of patients with prolactinoma don’t have to take DAs, even hyperprolactinemia relapses.

Among included studies, tumors were all shrank significantly (more than 50%) after DAs treatment. Generally, the initial tumor size affects the efficacy of treatment, the larger the tumor size is, the worse the efficacy is. In our study, the tumor diameter has none effects on the drug effects. In addition, our study showed that BRC and CAB have similar effects on controlling the relapse of prolactinomas. Finally, treatment duration of DAs were more than 24 months in all the studied included, which implicated that longer duration of normal prolactin level may help to decrease the possibility of tumor relapse. Ben-Jonathan N’s study indicated that DAs could inhibit the expression of prolactin gene and the proliferation of lactotrophs, and finally decrease the secretion of prolactin [24]. However, hyperprolactinemia is still recurred after DAs withdrawal, while the recurrent risk of prolactinoma is low. Compared with the tumor itself, the serum prolactin level seems to be more sensitive to the DAs withdraw, and the risk of hyperprolactinemia recurrence was very high. On the other hand, it is hard to determine that the risk of tumor relapse is low, until enough follow-up period is performed. Therefore, much more prospective studies with larger number of patients and longer follow-up period are warranted to further declare our finding.

In the analysis of heterogeneity source, meta-regression did not find the source of heterogeneity. Hence, difference among region of publication, year of publication, tumor size, drug type, treatment duration and follow up time did not cause heterogeneity. Funnel plot and Egger test found the presence of publication bias. Sensitivity analysis indicated three studies, Molitch’s study published in 1985, Sobrinho’s study published in 1981 and VanT Verlaat’s study published in 1991, were the source the heterogeneity. The recurrence proportions of prolactinoma after withdrawal of DAs in these three studies were 75% (3/4), 33.3% (3/9) and 25% (3/12), respectively [25–27]. These three studies were published before 2000 [25–27]. Long time gap may lead to changes in many aspects, such as the way the DAs was used, the purity of DAs, the manufacturer of DAs, the detect accuracy of prolactinoma and the guideline of treatment. Besides, the DAs used in these three studies were all BRC [25–27]. Compared with CAB, the way the BRC is used is more complicated due to shorter duration of action, which indicates the poorer compliance of BRC. In Sobrinho’s study, the follow up time was relatively shorter than other studies [26]. Therefore, synthesis of results was corrected by excluding these three studies. Through a serial of analysis including I^2^ test, χ2 statistic, meta-regression, sensitivity analysis, Egger test and funnel plot, we reduced heterogeneity significantly and obtained a relatively satisfied robustness. Ultimately, the heterogeneity among included studies were low, and results were creditable.

Our study also has some limitations. Firstly, there were only a few studies explored the relationship between recurrent prolactinoma and withdrawal of DAs directly. Secondly, the data extracted were mostly the part of results of the studies discussed the relationship between recurrent hyperprolactinemia and DAs withdrawal. Additionally, some retrospective studies included in the present study failed to give detail information about the treatment duration, which is a vital prognostic factor for the treatment of DAs on prolactinomas. What’s more, the publication bias was presented in this systematic review and meta-analysis. Therefore, more studies related to recurrent risk of prolactinoma after withdrawal of DAs should be done in future.

## Conclusion

Our study showed a very low recurrent rate of prolactinomas after DAs withdrawal, regardless of initial tumor size and type of DAs used. Much more prospective studies with larger cases and longer follow-up period are encouraged to confirm our finding.

## Data Availability

Not applicable.

## Abbreviations

DAs: dopamine agonists
CI: confidence interval
BRC: bromocriptine
CAB: cabergoline
PRL: prolactin
CT: computed tomography
